# S4-5 Framework for evaluation of green spaces for physical activity to support public health policy in Slovenija

**DOI:** 10.1093/eurpub/ckad133.022

**Published:** 2023-09-11

**Authors:** Ina Šuklje Erjavec, Jana Kozamernik, Vita Žlender, Simon Koblar

**Affiliations:** The Urban Planning Institute of the Republic of Slovenia; The Urban Planning Institute of the Republic of Slovenia; The Urban Planning Institute of the Republic of Slovenia; The Urban Planning Institute of the Republic of Slovenia

## Abstract

**Purpose:**

Evidence suggests that green space use for physical activity significantly benefits to maintenance and improvement of public health. Settlements need to provide adequate access to public green spaces which enable and encourage physical activity. Provision of publicly accessible green spaces is therefore often included in different sectoral strategies and policies. However, to really meet goals and objectives, declared in these documents, appropriate and efficient actions need to be taken in spatial development practice on a local level.

**Methods:**

We present the approach, developed within the first year of the national research project PREZENCA, which aims at developing indicators for the assessment of the provision of settlements with green spaces for outdoor physical activity in Slovenian municipalities. The approach may be of interest to countries which have adequately developed strategic spatial planning of urban green spaces but lack useful tools to assess the current state and to monitor changes in the provision of quality green and other outdoor spaces to encourage physical activity.

**Results:**

The approach is based on a comprehensive analysis of examples and possibilities for measuring presence and quantity of green spaces, defining green space types, and measuring citizens’ accessibility and use of green spaces for physical activity. Content analysis of existing planning documents on a local level was carried out to elaborate: (1) whether the provision of green spaces in settlements in Slovenia is defined and how (2) which population groups are addressed and (3) where such content should be put into policy documents to support implementation in practice. Based on the results, the suggestions on relevant factors and indicators of green space provision and quality for physical activity were prepared to be further developed for evaluation and monitoring on the local level and included into spatial planning practice and policies.

**Conclusion:**

Furthermore, we discuss the importance of establishing local standards and possibilities to include them into planning documents to form the basis for long-term monitoring of the condition of publicly accessible green spaces for recreational use by residents.

